# Absence of the Celiac Trunk

**DOI:** 10.5334/jbsr.1732

**Published:** 2019-02-01

**Authors:** M. Van den broecke, B. Leenknegt, L. Delrue

**Affiliations:** 1 Department of Radiology, University Hospital Ghent, BE

**Keywords:** absent celiac trunk, celiac trunk, vascular variations

## Case Presentation

**Figure 1:**
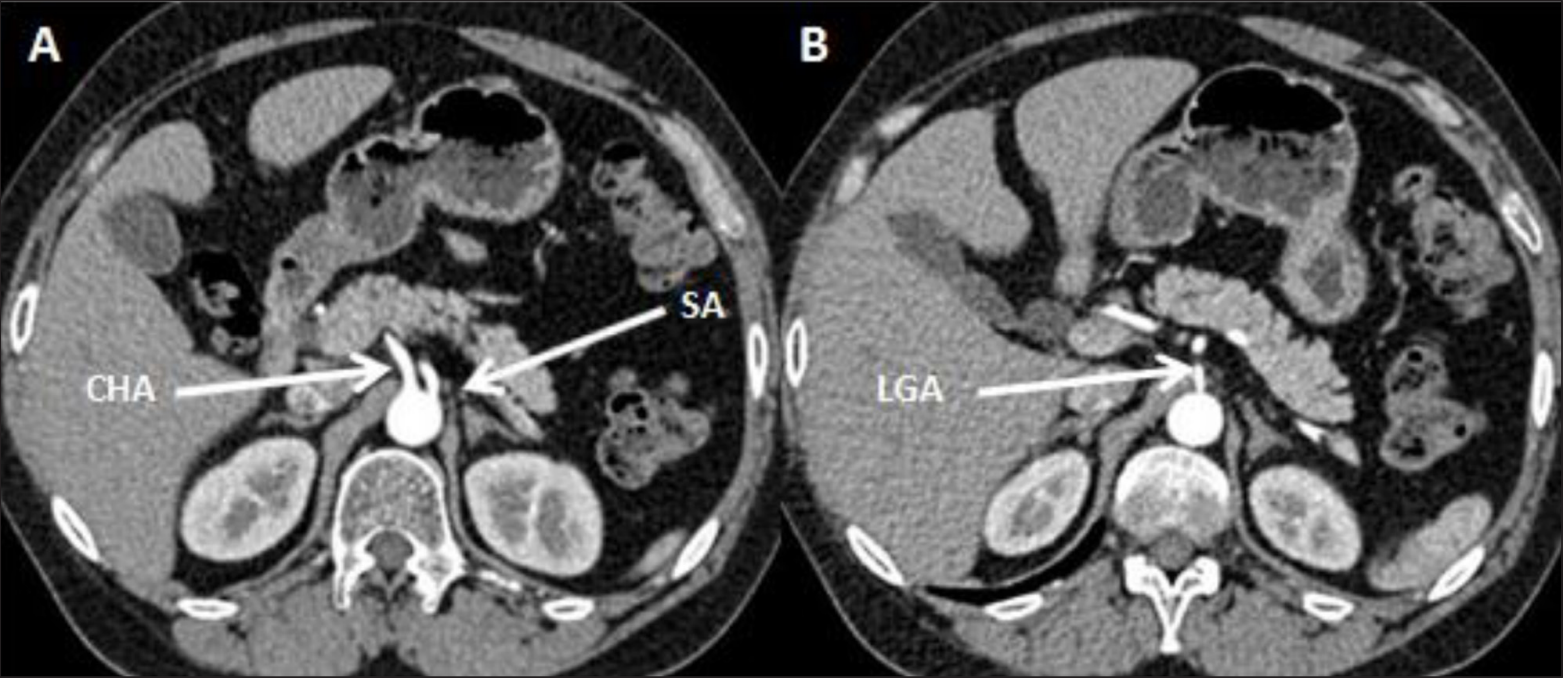
Abdominal CT scan in arterial phase demonstrate absence of the celiac trunk; the left gastric artery (LGA), splenic artery (SA), and common hepatic artery (CHA) arise directly and independently from the abdominal aorta.

**Figure 2:**
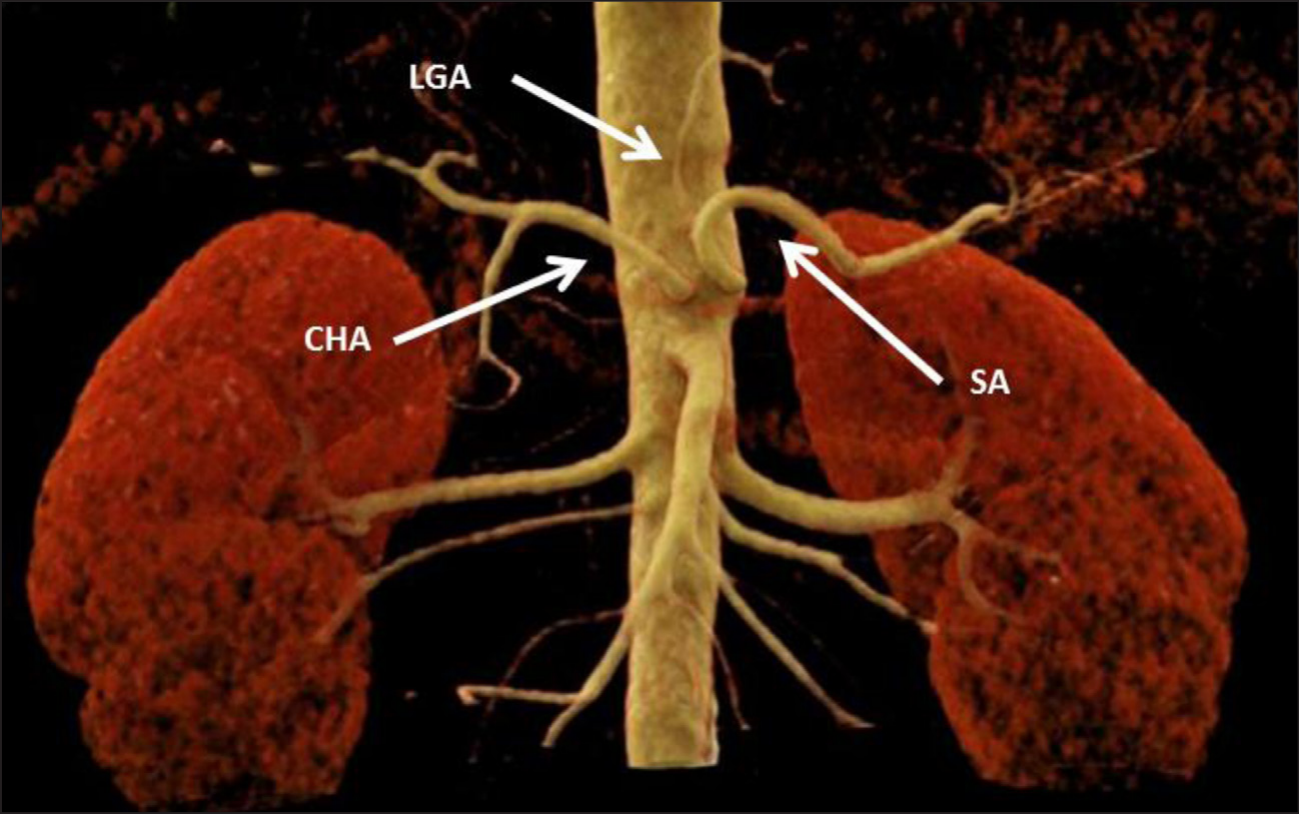
Volume rendering technique (VRT) of the celiac trunk; the left gastric artery (LGA), splenic artery (SA), and common hepatic artery (CHA) arise directly and independently from the abdominal aorta.

A patient with a history of Ewing sarcoma in the right thoracic wall was referred to the radiology department for a follow-up computed tomography (CT) scan. On CT, absence of the celiac trunk was noted. The left gastric artery, splenic artery, and common hepatic artery arose directly and independently from the abdominal aorta (Figures 1 and 2).

## Comment

The celiac trunk is the artery of the foregut, the proximal part of the alimentary tract. It arises from the abdominal aorta at the vertebral level T12–L1 and extends approximately 1.5–2 cm before dividing into three branches: the left gastric artery, the common hepatic artery, and the splenic artery. Absence of the celiac trunk is defined as the anatomical variant in which the left gastric artery, splenic artery, and common hepatic artery arise directly and independently from the abdominal aorta. It was first described by Saint Hilaire in 1832 and is considered a normal anatomical vascular variant. In a series of 10750 CT scans, an absent celiac trunk was found in only 0.19% of cases. Anatomical variations of the celiac trunk result from developmental changes in the ventral splanchnic arteries. The splanchnic arteries are formed by the fusion of the paired ventral segmental arteries. It is believed that the segmental arteries are united by “longitudinal anastomoses” to form the celiac trunk. Regression of the longitudinal anastomoses and persistence of the root of the segmental arteries would subsequently result in an absence of the celiac trunk. The classification system by Morita, based on embryological studies by Tandler, was the first to include an absent celiac trunk as a morphological subtype. Five variant types of the celiac trunk were proposed: the celiac trunk, hepatosplenic trunk, gastrosplenic trunk, hepatogastric trunk, and absent celiac trunk. Knowledge and correct description of the anatomical variants of the celiac trunk is important for clinical practice, as it can affect surgical approaches or endovascular procedures in this region [1].
